# Immunomodulatory potential of *Sarcophaga argyostoma* larval hemolymph as a natural alternative to berenil in treating *Trypanosoma evansi* in vivo

**DOI:** 10.1038/s41598-024-57113-y

**Published:** 2024-03-23

**Authors:** Al-Shaimaa M. Sadek, Doaa S. Farghaly, Hala Kadada, Alya Mashaal

**Affiliations:** 1 Parasitology, Zoology and Entomology Department, Faculty of Science (for Girls), Al-Azhar University, Cairo, Egypt; 2 Medical Entomology, Zoology and Entomology Department, Faculty of Science (for Girls), Al-Azhar University, Cairo, Egypt; 3 Taxonomy, Zoology and Entomology Department, Faculty of Science (for Girls), Al-Azhar University, Cairo, Egypt; 4 Immunology, Zoology and Entomology Department, Faculty of Science (for Girls), Al-Azhar University, Cairo, Egypt

**Keywords:** Immunology, Zoology

## Abstract

This study compared effects of diminazene aceturate (berenil), commonly used to treat domestic animals infected with *Trypanosoma evansi,* with the hemolymph of *Sarcophaga argyostoma* larva. The hemolymph may be acting as a possible natural alternative to berenil, based on immunomodulation mediated inflammatory response. Inflammatory mediators and histopathological changes in liver, kidney, and spleen of albino mice experimentally infected with *T. evansi* were studied. Mice were divided into five groups: G1, uninfected, untreated (negative control); G2,* T. evansi* infected (positive control); G3, infected and treated with berenil; G4, infected and treated with hemolymph; G5, infected and treated with hemolymph 3 days before infection (prophylactic group). Animals in (G4) and (G5) exhibited a significant overall reduction in serum levels of IFN-γ. However, the reduction in TNF-α and IL-6 levels was more limited compared to (G2) and (G3). Notably, an elevation in IL-10 levels was observed compared to animals in other groups. Furthermore, the groups treated with hemolymph demonstrated an alleviation of *T. evansi* infection in contrast to the other groups. This study highlights that the administration of *Sarcophaga argyostoma* larval hemolymph at a dosage of 0.5 ml/kg significantly inhibited *T. evansi* organisms in vivo, showcasing a pronounced trypanocidal effect.

## Introduction

Trypanosomiasis is a common and serious disease in several camel breeding countries^1^. The disease is common and results in significant economic loss throughout tropical and subtropical regions of the world, including Asia, Africa, the Middle East, Central America, and South America. Affected animals become listless, lose strength, weight, and often die without veterinary intervention^2^. Chemotherapeutic agents are a principal method of treatment, as investigation of vaccines for African animal trypanosomiasis is still in progress^3^. Trypanosomiasis causes immunosuppression responsible for loss of host capacity to eradicate trypanosomes even after administration of chemical therapy^4^. Chemical agents, such as diminazene aceturate and isomethamidium chloride, are currently used as trypanocides, both for prophylaxis and treatment of infected animals^5^. Unfortunately, *Trypanosoma* sp. display progressing resistance to these agents^6,7^_._ An effective alternative has been sought among natural products to fill the impending void in effective drugs.

Many natural products are used medicinally throughout the world^8^. Numerous substances isolated from insects have been examined in search of new therapeutic agents^9^. Lacerda et al.^10^ identified peptides from insects with activity against three endemic parasites: *Trypanosome*s, *Plasmodium* sp., and *Leishmania*. Moreover, future applications were discussed for use of insect peptides as anti-parasitic drugs and exploration of non-hosts insect transcriptomes in the quest for novel molecules for the treatment of parasitic diseases^10^.

Natural products from many insect species, such as moths, cockroaches, termites, flies, ants, bees, wasps, and beetles, and more, have been isolated. Biologically, these compounds exhibit antioxidant, antimicrobial, antifungal, antiviral, anticancer, immunomodulatory and anti-inflammatory properties^11^.

Several studies indicate that dipterous insects live in dirty environments saturated with microbes. These insects seemingly must possess vigorous cellular and humoral immune components to counter infection^12^. These compounds, in insect bodies, eggs, and body fluids, may show therapeutic efficacy as antimicrobial, anticancer, anti-inflammatory agents. Some substances have been used in folk medicine to treat such diseases^13^. Flesh flies are an important family of the order Diptera because of their impact on medical and veterinary science^14^. Insects are known for countering microorganisms by the release of antimicrobial substances, e.g., peptides into their body fluids^15^.

Antibacterial peptides were isolated more than thirty years ago from the hemolymph of *Hyalophora cecropia* pupae, and from female reproductive accessory glands of* Ceratitis capitata* and *Sarcophaga peregrina* larvae^16–18^. In folk medicine, maggots of *Lucilia sericata* (Meigen) (Diptera, Calliphoridae) are employed for maggot therapy. This therapy remains suitable for cases where antibiotics are ineffective and surgery impractical^19^. Medicinal maggots are reported to possess antibacterial, immunoactive, antiviral and antitumor properties^20^. Several investigations also studied antibacterial and immunoactive materials in *Musca domestica* maggots, such as prophenol oxidase, antibacterial protein/peptide, lysozyme and other secretions^21^. Shittu et al.^22^ investigated antiplasmodial activity of crude methanolic extracts of *Musca domestica* maggots in *Plasmodium berghei* infected mice and suggested that insects and their extracts suppress levels of parasitemia. Such an extract or its components might be developed as a natural product for treatment of malarial and other parasitic diseases. So, this study aims to use hemolymph of *Sarcophaga argyostoma* larva as a possible natural alternative to berenil, based on immunomodulation mediated inflammatory response.

## Methods

### Ethical approval

This study was approved by the Ethics Committee at Faculty of Medicine (Girl’s branch, Al-Azhar University (No: 2023031868, 18 July 2023). The experiment adhered to the National Institutes of Health's guidelines outlined in their publication (NIH Publication No. 85-23, revised 1996) regarding the care and utilization of laboratory animals. Additionally, the experiment's structure met the standards set by the ARRIVE criteria.

### Insect colony

A piece of fresh meat was placed in an open wooden box to start the stock colony of *S*. *argyrostoma* flies, The final larval instars of *S. argyrostoma* were transferred to 40 × 40 × 70 cm^3^ hard plastic cages, where they would pupate. The culture was maintained at the insectary of the Zoology and Entomology Department, Faculty of Science, Al-Azhar University (Girl's branch), at controlled conditions (25 ± 5 °C; L14: D10; 60 ± 5% RH). While the adults were fed sugar and water (1:2), the larvae were grown on beef steak^23^. Fresh or frozen meat was put to a Petri dish and changed daily to serve as a larviposition medium. The adult cages were inspected in the morning once a day.

### Preparation of hemolymph of *Sarcophaga argyrostoma* larva

Following CO2 anesthesia, the anterior tips of 50 larvae per sample were clipped with sterile, fine scissors to utilize the hemolymph, and then placed in an ice-cold Eppendorf with a few crystals of phenylthiourea to block the melanization process^24^. The hemocytes and hemolymph were separated by centrifugation for 10 min at 1000*g* and 4 °C^25^. The supernatant was then stored at − 20 °C until it was needed.

#### Gas–liquid chromatography (GLC) analysis for carbohydrates

Chromatographic analysis using GLC was performed by the Thermo system (Ultimate 3000) to analyze and detect carbohydrates, phenolics and fatty acids. The system comprised a pump, automatic sample injector, and a DELL-compatible computer running the Cromelion7 interpretation program. Detection was carried out using a RefractoMax520 refractive index detector. An aminopropyl-bonded phase column (4 gm high-performance carbohydrate column, waters) running at 30º C was used for the analysis. An isocratic solution of acetonitrile and water (75:25) made up the mobile phase. To reduce interference from NaCl, sodium chloride was added at a concentration of 0.125% (w/v)^26^.

#### Gas–liquid chromatography (GLC) analysis for phenolics

A diode array detector (DAD-3000) was used to measure phenolics. The Thermo-hypersil reversed phase C18 column, measuring 2.5 × 30 cm, operated at 25 °C. The mobile phase comprised 0.05% trifluoroacetic acid/acetonitrile (solvent A) and distilled water (solvent B). UV absorption spectra for both standards and samples were collected within the 230–400 nm range. Prior to analysis, the mobile phase, standard solutions, and samples were degassed and filtered through a Millipore 0.45 µm membrane filter. Compound identification was achieved by comparing their retention times and UV absorption spectra to those of the standards^27^.

#### Gas–liquid chromatography (GLC) analysis for fatty acids

The extracted fatty acids from both the sample and the standards were converted into the corresponding methyl esters using an ethereal diazomethane solution. Subsequently, the methyl esters of the fatty acids were analyzed using a GCV Pye-Unicam gas chromatography equipment. The proportions of the methyl esters of fatty acids were determined using a GLC column^28^.

#### Preparation and identification of amino acids by using amino acid analyzer

The SYKAM high-performance analyzer was employed for amino acid analysis and identification. Defatted hemolymph solution was obtained by three cycles of boiling under reflux with 50 ml of 50% ethanol, yielding one milliliter each time over three hours. To clarify the mixed ethanolic solutions, they were filtered and treated with a 10% trichloroacetic acid solution. The resulting supernatant was concentrated to 5 ml under reduced pressure and cleansed with distilled water. The filtrate was adjusted to a volume of 100 ml using distilled water. Five milliliters of the diluted sample were vacuum-dried at 70 °C and subsequently dissolved in a five-milliliter loading buffer (pH 2; 0.2 N sodium citrate buffer). After filtration through a 0.45-micron pore filter, the substance was introduced into the amino acid analyzer^29^.

Peaks were identified by comparing the relative retention times of each compound with those of reference materials. Following Farag's et al.^29^ method, the ratio of each compound's partial area to its total area was utilized to estimate their respective proportions.

### *Trypanosoma evansi* isolate

*T. evansi* was originally isolated from a camel naturally infected and raised in a rural area in the Assuit province in upper Egypt (27° 11′ N 31° 10′ E). Thin blood films were prepared from ear vein, dried, fixed in absolute methyl alcohol and stained with Giemsa stain according to Soulsby^30^^.^

### Experimental animals

Forty male albino mice (*Mus domesticus*) with average weight 35–40 g were obtained from Veterinary Collage, Assuit University, Egypt. Animals were examined and acclimated for 10 days before use. The animals were housed in standard laboratory conditions, maintained at 21°C with 16% moisture, provided ad-libitum access to water, and fed a diet consisting of 20% protein, 3% fat, and 22% fiber. Following experimental infection with *T. evansi*, blood samples were taken daily from day 4 to day 10 until the animals were sacrificed.

Groups (8 mice/group) were divided randomly as:

Group 1 (G1): uninfected, untreated (negative control).

Group 2 (G2): infected, untreated (positive control).

Group 3 (G3): Infected and treated with a single dose of 0.5 ml/kg diminazene aceturate (berenil) upon detection of the parasite in the blood.

Group 4 (G4): Infected and treated with a single dose of 0.5 ml/kg hemolymph upon detection of the parasite in the blood.

Group 5 (G5): Infected after three days of treatment with 0.5 ml/kg hemolymph (prophylactic group).

### *Trypanosoma evansi* inoculation

All animals in G2, G3, G4, and G5 were inoculated intraperitoneally with 0.3 ml of fresh blood, obtained from camels previously infected with *T. evansi*. The dose was approximately 10^4^ parasites/animal.

### Berenil preparation and administration

Drugs were dissolved and prepared as aliquots for intraperitoneal (I.P.) injection, following manufacturer's instructions. The dose of diminazene aceturate (Berenil, Hoechst, Germany) was 0.5 ml/kg of body weight according to MSD animal health instructions, 2020.

### Daily follow up and sampling

Daily examination in mice was checked. 1st, 2nd, and 3rd days no trypanosomes were appeared in the blood of infected mice (First three days considers prepatent period), the detection of parasitemia and counting number of trypanosomes was recorded from 4th day until sacrifice at the end of the experiment (on day 10 post infection) by cutting the tip of tail and collecting a drop of blood on clean glass slides. Blood was then examined under a microscope at 100 × according to Pizzi-Brener^31^. Trypanosomes in thin blood film were counted according to Kolmer method^32^. Upon completion of the experiment, blood was collected from the ocular vein, and serum was prepared for cytokines analysis. Animals were dissected immediately for histopathology preparation.

### Inflammatory mediators’ assessment

Cytokine quantification was measured following the manufacturer’s instructions, IFN-γ (RayBio® Mouse IFN-gamma ELISA Kit, Catalog #: ELM-IFNg); TNF-α (RayBio® Mouse TNF-alpha ELISA Kit, Catalog #: ELM-TNFa); IL-6 (RayBio® Mouse IL-6 ELISA Kit Catalog #: ELM-IL-6); IL-10 (ELISA assay using Ray Bio® ELISA Kits, Catalog #: ELM-IL-10). The concentration of the cytokines was determined by measuring color intensity at 450 nm using a microplate reader.

### Histopathological preparation and analysis

Samples of liver, kidney, and spleen were washed in saline solution and fixed in 10% formalin for 24–48 h at room temperature, embedded in paraffin, and cut into 4–6 µm sections. Sections were stained with hematoxylin–eosin stain using standard histological techniques^33^. Histopathological examination of tissues uses an Olympus BX51 light microscope.

### Statistical analysis

Data were analyzed using the descriptive statistics procedure in the SPSS (IBM, USA, v23) statistical package. The difference between means was evaluated by one-way analysis of variance (ANOVA) followed by post hoc test (LSD). Means values were calculated for all treatments. The least significant difference method was used for comparing means. Differences among means with *p* < 0.05 were accepted as statistically significant, and differences of 0.05 < *p* < 0.10 were accepted as tendencies.

## Results

### Determination of chemical compounds using gas–liquid chromatography (GLC) and amino acid analyzer in the hemolymph of *Sarcophaga argyostoma* larva

The chemical compounds, including carbohydrates, phenolics, and fatty acids, are listed in Table 1, with their identification based on parameters such as relative time (minutes), area (mAU*min), height (mAU), relative area (%), relative height (%), and amount (µg). The results indicate the presence of lactose and fructose, classified as carbohydrates (sugars), along with 5 phenolic compounds and 21 fatty acids. Additionally, the amino acid analysis in Table 2 revealed the presence of 17 compounds identified by retention time (min.), response amount (PPM), and amount (%).

**Table 1:** The names of chemical compounds found in the hemolymph of *Sarcophaga argyostoma* larva identified through gas–liquid chromatography (GLC).

No	Peak name	Retention time, min	Area, mAU*min	Height, mAU	Amount, mg	Chemical formula
Carbohydrates						
1	Lactose	2.053	31.374	1581.906	712.8729	C_12_H_22_O_11_
2	Fructose	2.300	337.509	2896.932	5171.3366	C6H12O6
Phenolic acids						
1	Quinic acid	2.537	1.658	28.031	8.2399	C_7_H_12_O_6_
2	Apegenin	2.477	1.090	28.783	8.0506	C_15_H_10_O_5_
3	Chlorgenic acid	2.633	1.306	18.105	9.0986	C_16_H_18_O_9_
4	Gallic acid	2.990	16.847	630.056	8.7597	C_7_H_6_O_5_
5	Cinnamic acid	3.370	6.091	71.634	8.3351	C_9_H_8_O_2_
Fatty acids						
1	Enanthic acid	2.835	1.53	0.96	1.3455	C_7_H_14_O_2_
2	Butric acid	3.273	1.31	1.00	1.6754	C_4_H_8_O_2_
3	Caproic acid	3.910	1.26	0.69	1.1083	C_6_H_12_O_2_
4	Caprylic acid	4.965	0.32	0.29	3.456	C_8_H_16_O_2_
5	Pentadecanoic acid	10.073	2.24	1.02	3.987	C_15_H_30_O_2_
6	Myristoleic acid	10.081	0.75	0.97	2.1613	C_14_H_26_O_2_
7	Myristic acid	10.094	1.26	0.83	3.452	C_14_H_28_O_2_
8	Stearic acid	13.810	40.52	10.60	19.519	C_18_H_36_O_2_
9	Oliec acid	13.820	77.32	10.57	19.420	C_18_H_34_O_2_
10	Linoleic acid	14.725	1.69	0.43	7.1019	C_18_H_32_O_2_
11	Linolenic acid	15.837	1.20	0.50	6.233	C_18_H_30_O_2_
12	gamma-Linolenic acid	15.995	0.69	0.33	5.768	C_18_H_30_O_2_
13	Arachidic acid	16.039	0.40	0.31	7.390	C_20_H_40_O_2_
14	cis-8,11,14-Eicosatrienoic acid	16.708	0.38	0.23	6.990	C_20_H_34_O_2_
15	cis-11,14,17-Eicosatrienoic acid	16.804	11.64	4.39	7.876	C_20_H_32_O_2_
16	cis-11,14-Eicosadienoic acid	16.821	3.26	4.08	5.556	C_20_H_34_O_2_
17	cis-11-Eicosenoic acid	16.830	2.20	3.89	4.765	C_22_H_38_O_2_
18	Linoleliadic acid	16.837	2.21	3.59	7.854	C_18_H_32_O_2_
19	Arachidonic acid	16.847	5.92	3.17	7.384	C_20_H_32_O_2_
20	cis-5,8,11,14,17-Eicosapentaenoic acid	17.173	0.39	0.19	8.989	C_20_H_30_O_2_
21	Heneicosanoic acid	17.367	2.19	0.68	7.987	C_21_H_42_O_2_

*min.* minute, *mAU* milli-absorbance units, *mg* milligrams.

**Table 2:** The amino acid names identified in the hemolymph of *Sarcophaga argyostoma* larva using an amino acid analyzer.

	Compound name	Retention time (min.)	Response	Amount (PPM)	Amount (%)	Chemical formula
1	Aspartic acid (ASP)	9.032	1281.622	3.391	7.0	C_4_H_7_NO_4_
2	Threonine (THR)	11.589	551.531	1.199	2.5	C_4_H_9_NO_3_
3	Serine (SER)	12.571	645.912	1.328	2.7	C_3_H_7_NO3
4	Glutamic acid (GLU)	15.363	2334.592	7.025	14.5	C_5_H_9_NO_4_
5	Glycine (GLY)	22.147	773.249	1.231	2.5	C_2_H_5_NO_2_
6	Alanine (Ala)	22.971	2051.006	3.841	7.9	C_3_H_7_NO_2_
7	cysteine (CYS)	23.403	703.927	2.580	5.3	C_3_H_7_NO_2_S
8	Valine (VAL)	24.587	772.829	1.843	3.8	C_5_H_11_NO_2_
9	Methionine (MET)	25.845	325.779	1.046	2.2	C_5_H_11_NO_2_S
10	Isoleucine (ILE)	28.755	498.769	1.423	2.9	C_6_H_13_NO_2_
11	Leucine (LEU)	29.717	1334.542	3.331	6.9	C_6_H_13_NO_2_
12	Tyrosine (TYR)	31.859	801.849	2.782	5.7	C_9_H_11_NO_3_
13	Phenylalanine (PHE)	32.989	1193.706	3.741	7.7	C_9_H_11_NO_2_
14	Histidine (HIS)	35.328	1425.788	4.743	9.8	C_6_H_9_N_3_O_2_
15	Lysine (LYS)	38.235	905.805	3.494	7.2	C_6_H_14_N_2_O_2_
16	Proline (PRO)	40.165	4411.565	3.941	8.1	C_5_H_9_NO_2_
17	Arginine (ARG)	41.637	374.773	1.463	3.3	C_6_H_14_N_4_O_2_

*min.* minute, *PPM* parts per million.

### Parasitemia estimation

**Figure 1:**
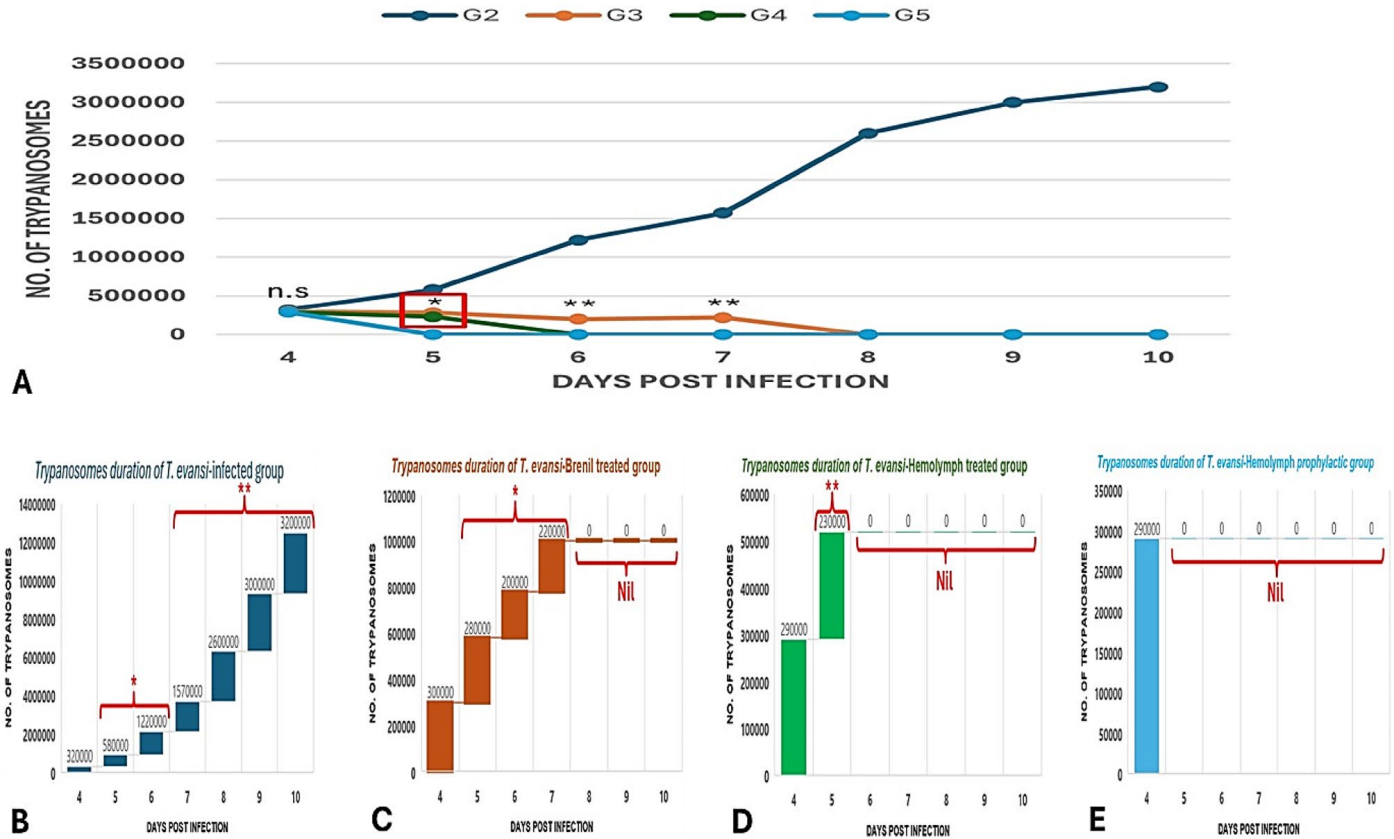
Trypanosomes daily progression within different groups. Values are expressed as parasitemia mean ± standard deviation (SD), n = 8 mice. (**A**) Comparative numbers compared to positive control group (T. evansi infected group); (**B**) Trypanosomes progression in positive control group; (**C**) Trypanosomes progression in Berenil-treated group; (**D**) Trypanosomes progression in hemolymph-treated group; (**E**) Trypanosomes progression in hemolymph prophylactic-group. G2: *T. evansi* infected; G3: berenil-treated; G4: hemolymph-treated group; G5: hemolymph-prophylactic group; Nil: not detectable. **High significant (P < 0.01); *significant (P < 0.05); n.s non-significant vs. positive control group (**A**), vs 4th day p.i (**B**–**E**).

The hemolymph of *Sarcophaga argyrostoma* larva was investigated as an a trypanocidal alternative for berenil. The concentration in this experiment was 10 mg/ml, and dose rate was 0.5 ml/kg BW. The average prepatent period ranged from 2–3 days. Trypanosomes are detected within all groups with average incidence/mm^3^ 32 × 10^4^ in G2, 30 × 10^4^ in G3, 29 × 10^4^ in G4 and 29 × 10^4^ in G5. All treated mice became All treated mice became aparasitemic at the end of the experiment with a clearance percentage of 100%. The time courses of parasitemia show initial clearance of parasites began on day six in G4 and day five in G5 post-infection, while in G3 mice, treated with berenil, parasites in blood decreased slowly before finally disappearing on day eight post-infection (Fig. 1).

### Inflammatory mediators

Immunomodulation of inflammatory cytokine levels in different treated, infected, and negative control after 10 days of infection showed in Table 3*.* Serum levels of* IFN-γ* decreased (*P* < 0.001) in G3, G4, and G5-treated groups respectively compared to both positive and negative control groups. An increase in the serum levels of TNF-α and IL-6 in infected and treated animals was observed compared to the -ve control group. In contrast, IL-10 serum level was reduced in G3 mice compared to the -ve control group but elevated in G4 and G5* c*ompared to the + ve and −ve control group respectively (Table 3)*.*

**Table 3:** The change of inflammatory mediator’ levels (pg/ml) in different infected and treated groups compared with control at the end of experiment.

Cytokine	Group				
	G1	G2	G3	G4	G5
IFN-γ (pg/ml)	28 ± 0.327	35 ± 0.432***	25 ± 0.245***^a^	20 ± 0.71***^ab^	15 ± 0.4***^ab^
TNF-α (pg/ml)	22 ± 0.4	35 ± 0.42***	26 ± 0.4***^a^	23 ± 0.20**^ab^	24 ± 0.432***^ab^
IL-6 (pg/ml)	10 ± 0.163	20 ± 0.779***	12 ± 0.216***^a^	10.6 ± 0.26 ^ab^	11 ± 0.283*^ab^
IL-10 (pg/ml)	7 ± 0.432	6 ± 0.163***	6.9 ± 0.082 ^a^	7.5 ± 0.41*^ab^	8.2 ± 0.216***^ab^

Values are expressed as mean ± standard deviation (SD), n = 8 mice.

*G1* negative control, *G2 T. evansi* infected, *G3* berenil-treated, *G4* hemolymph-treated group, *G5* hemolymph-prophylactic group, *IFN-γ* interferon-gamma, *TNF-α* tumor necrosis factor, *IL-6* interleukin-6, *IL-10* interleukin-10.

*P < 0.05; **P < 0.01; ***P < 0.001 vs. negative control group.

^a^P < 0.05 vs. positive control group (*T. evansi* infected group).

^b^P < 0.05 vs. berenil-treated group.

### Histopathological findings

#### Liver

**Figure 2:**
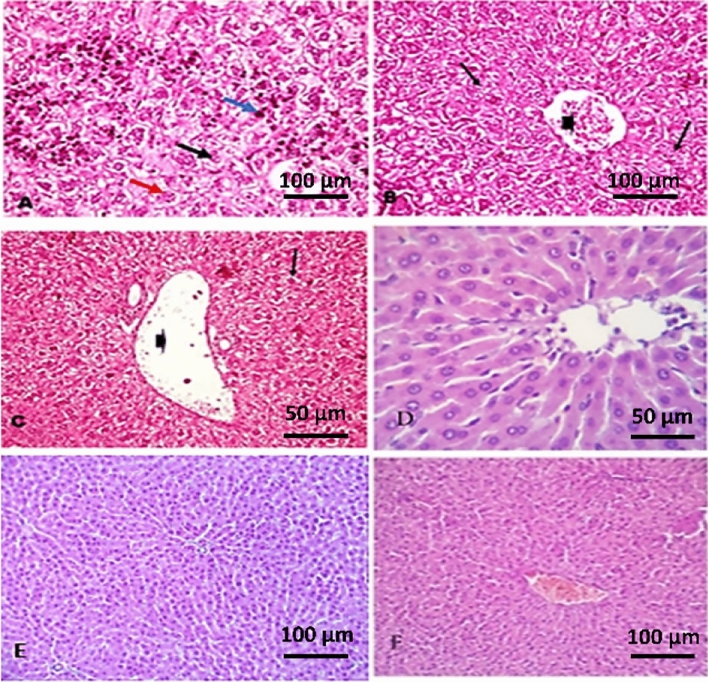
Photomicrograph of liver sections. (**A**) and (**B**) liver histology of mice in the positive control group (G2); (**A**) showing diffuse and massive degenerative changes including granular cytoplasm (red arrow), nuclei are either pyknotic (blue arrow) or vesicular (black arrow). (**B**) Liver showing congested central vein (arrowhead) with massive, degenerated hepatocytes (arrows). (**C**) Liver histology of G3 (infected and treated with berenil) showing dilatation of the hepatoportal blood vessels (arrowhead) and diffuse vacuolation of the hepatic parenchyma (arrows). (**D**,**E**) Livers of extract-treated groups before and after infection (G4, G5) mice showing normal liver histology. (**F**) Normal hepatocytes in negative control mice (G1). H&E.

Liver variably changes from moderate histopathological changes in G3 mice to severe degenerative changes G2 are shown in Fig. 2. Livers from mice in G2 that did not receive treatment showed hepatomegaly, diffuse and massive degenerative changes, including granular cytoplasm and pyknotic or vesicular nuclei (Fig. 2A). Significant congestion in central vein with massive, degenerated hepatocytes was also present (Fig. 2B). Livers from mice in G3, treated with berenil, showed dilatation of hepatoportal blood vessels and diffuse vacuolation of hepatic parenchyma (Fig. 2C). Livers of mice in G4 and G5, treated with hemolymph after and before infection respectively, maintained their original structure (Fig. 2D,E) as also observed in livers normal negative control mice (G1) (Fig. 2F).

#### Kidney

**Figure 3:**
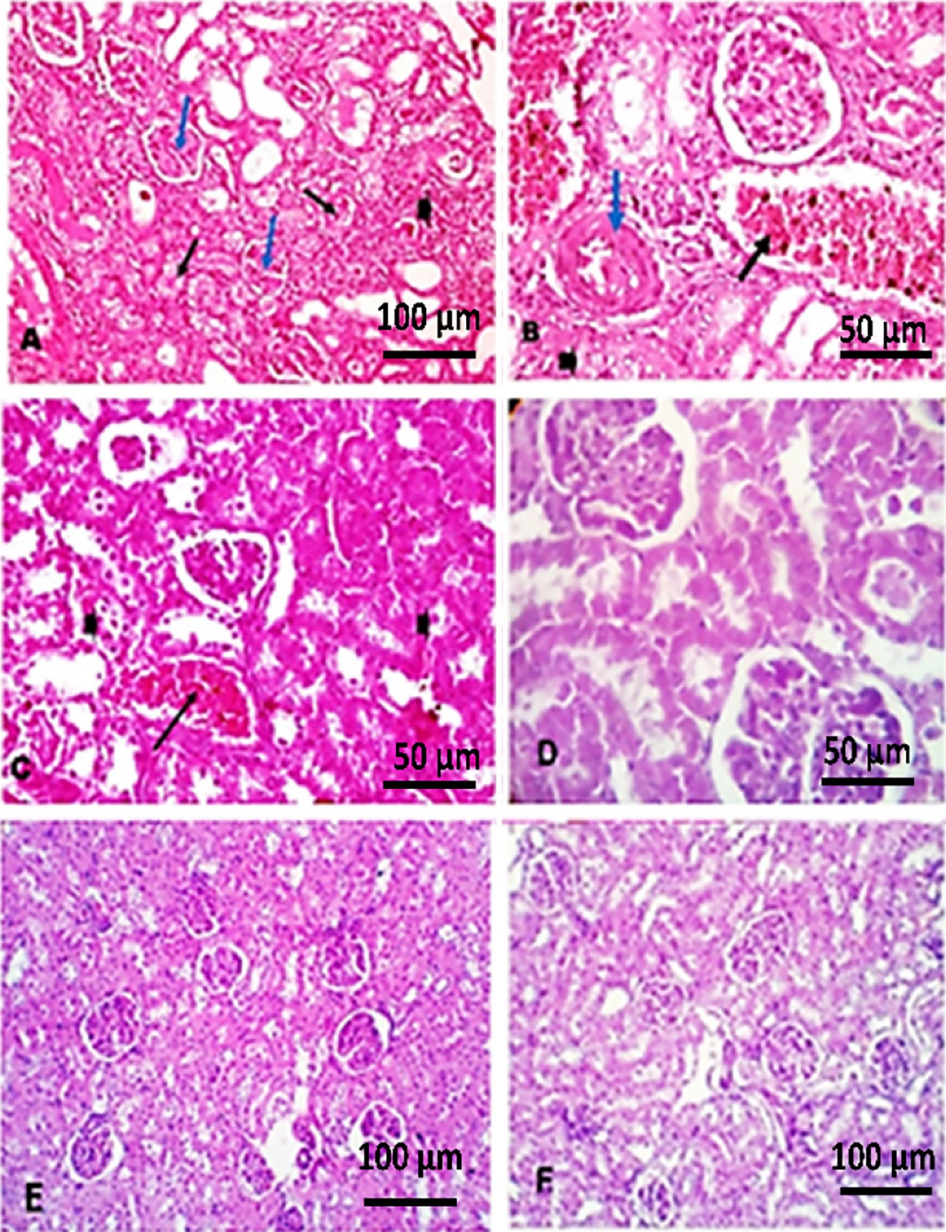
Photomicrograph of Kidney sections. (**A**) and (**B**) Kidney histology of mice in (G2) positive control group, (**A**) showing multi focal aggregation of mononuclear cells (arrowhead), together with degeneration in both renal tubules (black arrows) and glomeruli (blue arrow), (H&E × 200). (**B**) Kidney showing degenerated renal tubules (arrowhead), interstitial blood vessels showing severe congestion (black arrow) while some showing thickened muscular wall (blue arrow), (H&E × 400). (**C**) Kidney histology of mice infected and treated with berenil (G3) showing congestion in the interstitial blood vessels (arrow) with degenerated and disintegrated renal tubules (arrowhead), (H&E × 400). (**D**,**E**) Kidney histology of mice in (G4, G5) showing normal kidney structure, (H&E × 400). (**F**) Kidney histology of control negative group (G1). H&E.

The kidney histopathological changes are shown in Fig. 3. Mice in G2 showed multifocal aggregation of mononuclear cells and degeneration in both renal tubules and glomeruli (Fig. 3A), severe congestion in interstitial blood vessels and thickened muscular walls (Fig. 3B). Kidneys of mice from G3 showed congestion in interstitial blood vessels with degenerated and disintegrated renal tubules (Fig. 3C). The kidneys of mice in G4 and G5 retained nearly normal architecture (Fig. 3D,E) when compared with the negative control group (G1) (Fig. 3F).

#### Spleen

**Figure 4:**
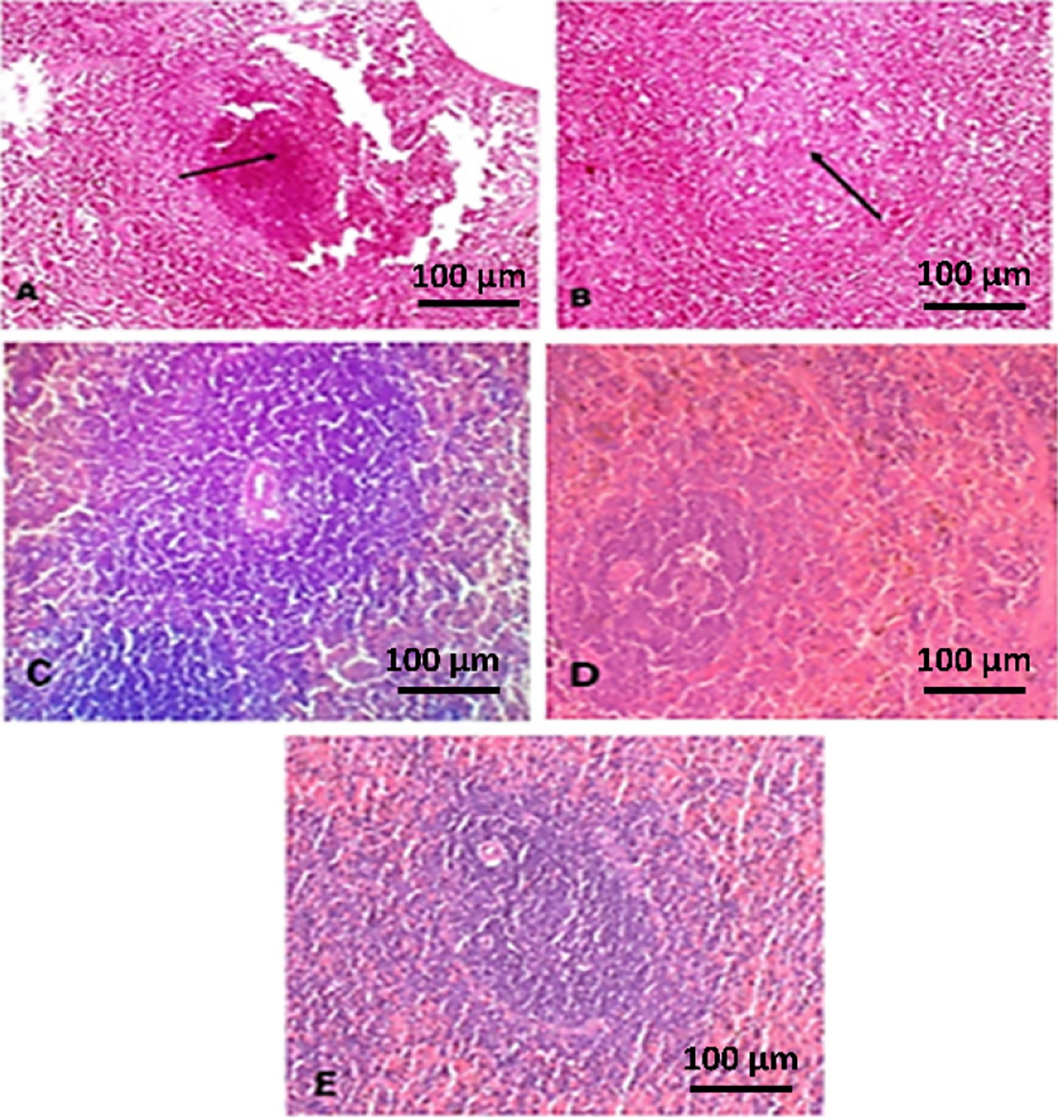
Photomicrograph of spleens. (**A**) shows necrosis in the splenic lymphoid follicles (arrow) in the infected untreated group (G2). (**B**) shows atrophy in some splenic lymphoid follicles (arrow) in the infected group treated with berenil (G3). (**C**,**D**) shows normal splenic cells in the infected group treated with extract (G4, G5) respectively. (**E**) Negative control group (G1). H&E.

The main histopathological features in the spleen are shown in Fig. 4. The spleen of the infected group (G2) consisted of necrosis in splenic lymphoid follicles (Fig. 4A) and atrophy in splenic lymphoid follicles in mice in G3 (Fig. 4B). Upon treatment with extract, spleen structure remained almost normal. The extract significantly spared histological features of the spleen (Fig. 4C,D). The spleen of negative normal control is shown in Fig. 4E.

## Discussion

Several discussions of natural insect products and their potential for development into drugs to treat *T. evansi* diseases are available. Propolis extract can prolong the life of rats infected with *T. evansi*^34^ and methanol extracts of *Musca domestica* larva display trypanocidal properties^35^. The use of *Sarcophage* larvae for treatment of certain bacterial infections is years old. Okada and Natori^36^ purified three antibacterial proteins from the hemolymph of *Sarcophaga* larvae.

In this study chromatographic results showed that the hemolymph of *S. argyostoma* larvae is a valuable resource rich in phenolic, fatty, and amino acids. The presence of phenolic content in the hemolymph was identified and quantified by the analytical method of Gas–Liquid Chromatography (GLC). The extracts revealed the presence of phenolic acids such as quinic acid, apigenin, chlorgenic acid, gallic acid and cinnamic acid. Júnior et al.^37^ detected quinic acid, chlorogenic acid, and gallic acid in the leaf extract of *Guazuma ulmifolia*, a natural product that exhibits significant antiparasitic properties. Additionally, according to other study findings, specific phenolic substances in plant extracts possess the ability to inhibit kinetoplastid species such as *Leishmania* and *Trypanosoma*^38^.

As per the analysis of fatty acids, the chromatographic findings revealed the presence of 21 fatty acids. Hirazawa et al.^39^ suggested that short and medium-chain fatty acids (C4-C10) such as butyric acid, caproic acid, enanthic acid, and caprylic acid exhibit antiparasitic effects against *Cryptocaryon irritans* ciliates. Additionally, Goodman and McFadden^40^ demonstrated that synthesis inhibitors incorporating fatty acids offer a promising avenue for developing new anti-parasitic compounds.

One of the primary causes of insects' remarkable capacity to adapt to survive is the fact that antimicrobial peptides are mostly produced in the fat bodies and blood cells of insects^41^. Our study revealed the presence of 17 amino acids that belong to antimicrobial peptides (AMPs), found in amino acid-rich species^42^, such as glycine, arginine, proline, and histidine. Antiparasitic peptides demonstrate their ability to combat diseases like malaria and leishmaniasis^43^. Specific peptides, including lysine, aspartic acid, glutamic acid, and leucine derived from bee royal jelly, have exhibited considerable efficacy against the *Leishmania* parasite^44^.

Common problems with the use of berenil for the treatment of *T. evansi* infection are toxicity at therapeutic doses, including tachycardia and CNS signs such as ataxia, nystagmus, and opisthotonos^45^. Parasitemia significantly increased in G2 mice (positive control) while significantly reduced in the mice treated with berenil and hemolymph. The main difference between these two treatments is that mice treated with berenil became aparasitemic on the 8th day after infection, while mice treated with hemolymph were aparasitemic on the 5th day post-infection. These results agree with the previous study of Shittu et al.^35^, who recorded a significant decrease in* T. brucei* parasitemia in mice treated with 400 mg/kg of a methanolic extract of *Musca domestica* larvae.

The three primary proinflammatory cytokines associated with parasite infection—IFN-γ, TNF-α, and IL-6—have been identified. Research has shown that the anti-inflammatory cytokine IL-10 functions to counteract the effects of these proinflammatory cytokines, contributing to the maintenance of immunological homeostasis and regulation of inflammatory responses. In this study, IFN-γ showed a reduction in extract-treated groups compared to all different groups, also TNF-α and IL-6 significantly decreased in comparison with other treated and infected animals, IL-10 showed higher values than other different groups.

IFN-γ plays a crucial function in identifying and getting rid of parasites by mediating host–pathogen interactions. The initiation of a cascade of pro-inflammatory responses is caused by a complex interplay between immune cell activity and IFN-γ through coordinated integration of signals from other pathways involving cytokines and Pattern Recognition Receptors (PRRs) such as Interleukin (IL)-4, Lipopolysaccharide (LPS), TNF-α, IL-6 as in trypanosomiasis^46^.

INF-γ levels in the serum may be associated with resistance to parasitemia and regulation of the condition by activating macrophages, which eliminates the parasite from the bloodstream. Several authors have described this pathway for trypanosomiasis^47–49^.

TNF-α exhibits trypanolytic properties linked to elevated levels during trypanosomiasis infection^50,51^. This elevation potentially aids in controlling parasitemia, as observed by a subsequent decrease in bloodstream parasites following increased TNF-α levels. The inability to effectively manage parasitemia was associated with excessive production of proinflammatory cytokines, including IFN-γ and TNF-α, due to impaired activation of alternatively activated macrophages^52^. While the production of IFN-γ and TNF-α is crucial for protection, excessive production is detrimental, rendering infected mice more susceptible and leading to their death^53^_._

An increase in IL-6 was also described in mice infected with *T*. *brucei*^54^. IL-6 deficient was more susceptible to *T. cruzi*, infection and increased mortality rates^55^.

IL-10 has been shown to have anti-inflammatory properties to protect hosts from a number of parasitic diseases, such as trypanosomiasis^56^. IL-10 acts to downregulate excessive effector activities of both T cells and macrophages^53^ that are linked to protection. Sternberg et al.^54^ (2005) and Baldissera et al.^45^ reported that infection with *T. brucei* in a murine model showed upregulation of IL-10 levels and showed only mild inflammatory pathology. This finding is consistent with the present results in mice treated with hemolymph that showed no pathological changes.

In fact, protection during infection with trypanosomes (lower parasitemia and prolonged survival) is associated with the ability to switch from Th1 to Th2 response during the later stages of infection^52^. In line with this, the reduction of IFN-γ, TNF-α, and IL-6 signaling (which are key cytokines that drives Th1 differentiation), and elevation of IL-10 (which is a key cytokine that drives Th2 differentiation) that observed in hemolymph- treated trypanosomiasis mice of this study, led to the ability to control parasitemia compared to other counterpart mice.

During the initial examination of organs, hepatomegaly, observed in this study, is compatible with Brown and Losos^57^. The most drastic changes in livers were recorded in G2, followed by G3. G2 mouse livers showed massive degenerative changes in hepatocyte nuclei and cytoplasm possibly caused by consumption of oxygen by proliferating trypanosomes. Such consumption could deprive tissues of oxygen, resulting in degenerative changes^58^. The congestion and dilation in blood vessels observed in livers of G3 mice may be caused by hypoglycemia leading to starvation of host cells or perhaps to anoxia due to anemia caused by *T. evansi*^58^. Another explanation, suggested by Derakhshanfar et al.^59^ who reported that dilation of focal areas and congestion in sinusoids caused by accumulation of lipids inside liver cells due to tissue hypoxia, resulted from anemia, and vascular damage. The present results are also in agreement with many authors who reported fatty degeneration in hepatocytes of rats infected with *T. evansi*^57,60,61^.

Results observed in mice treated with berenil are in agreement with Homeida et al.^62^ who reported that berenil caused hemorrhage and degeneration in liver cells of camels infected with *T. evansi.* Adeyemi and Sulaiman^63^, also reported inflammation and congestion in hepatic and renal cells in rats infected with *T. brucei* and treated with berenil. These authors also mentioned elevation in alkaline phosphatase, alanine transaminase and aspartate transaminase from degenerative changes in hepatic cells.

Protective effects of hemolymph for histological structures of vital organs is consistent with Al-Otaibi et al.^64^ who showed that methanolic extracts of natural products, such as from *Lepidium sativum* seeds, can reduce hepatotoxicity, and limit damage to hepatocytes. These extracts resulted in significant improvement and normalization of liver enzyme levels.

This study also showed that infection with *T. evansi* in control group mice and mice treated with berenil resulted in severe congestion in interstitial blood vessels, multifocal aggregation of mononuclear cells, degeneration in renal tubules, degeneration and infiltration in renal cells may be caused by invading trypanosomes^63^ or perhaps by utilization of glucose and oxygen during parasite proliferation leading to deformities in kidney and liver^65^. Homeida et al.^62^ also reported the same changes in the kidneys of camels infected with *T. evansi*. The present results are also in agreement with Mbaya et al.^66^ who noticed various degrees of degenerative changes with cellular infiltrations in kidneys of gazelles infected with *T. brucei* and treated with diminazene aceturate at 3.5 mg/kg body weight. Results from G4 mice are supported by results of many authors who reported that natural products extracted from plants and insects and used as anti-trypanosomal drugs protect histological structures, and biochemical and enzyme systems of infected animals^34,45,67,68^.

Changes in spleen indicate a protective immune response correlated with inflammatory responses to infection. The inflammatory response may be the initial host response against protozoal infection or may be due to reduction in IL-10 levels^45,55^. Changes in splenic cells may be due to hypersensitivity to infection with *T. evansi*^58^ or to a response of *T. evansi* to its toxic metabolites that results in varying degrees of anemic anoxia and splenic damage^69^. Study results are in agreement with many authors who report hemorrhage and focal necrosis in spleens of donkeys and rats^60,69^ and with Morrison et al.^70^ who reported the same changes in dogs infected with *T. brucei*.

## Conclusion

This study marks the pioneering investigation into the chemical composition of *Sarcophaga argyostoma* larval hemolymph. Through chromatographic analysis, several valuable compounds were unveiled, showcasing potential for the refinement of therapeutic and pharmaceutical approaches in combatting parasitic and other related diseases. Notably, administration of *Sarcophaga argyostoma* larval hemolymph at a dosage of 0.5 ml/kg demonstrated a significant inhibition of *T. evansi* organisms in vivo, underscoring a robust trypanocidal effect. Moreover, hemolymph was found to modulate inflammatory mediators while exhibiting no adverse impact on immune system organs, such as the spleen. Furthermore, it conferred protective effects on vital organ structures, such as the liver and kidney, safeguarding them from damage induced by *T. evansi* infection. The administration of a prophylactic dose to potentially exposed animals within the region of risk showcases promising prospects in either preventing infection or bolstering the health of susceptible animals.

## Data Availability

Data will be available when requested from the corresponding author.
